# Discovering Weighted Patterns in Intron Sequences Using Self-Adaptive Harmony Search and Back-Propagation Algorithms

**DOI:** 10.1155/2013/249034

**Published:** 2013-05-08

**Authors:** Yin-Fu Huang, Chia-Ming Wang, Sing-Wu Liou

**Affiliations:** ^1^Department of Computer Science and Information Engineering, National Yunlin University of Science and Technology, 123 University Road, Section 3, Douliu, Yunlin 640, Taiwan; ^2^Graduate School of Engineering Science and Technology, National Yunlin University of Science and Technology, 123 University Road, Section 3, Douliu, Yunlin 640, Taiwan; ^3^Supercomputing Research Center, National Chen Kung University, 1 University Road, Tainan, 70101, Taiwan

## Abstract

A hybrid self-adaptive harmony search and back-propagation mining system was proposed to discover weighted patterns in human intron sequences. By testing the weights under a lazy nearest neighbor classifier, the numerical results revealed the significance of these weighted patterns. Comparing these weighted patterns with the popular intron consensus model, it is clear that the discovered weighted patterns make originally the ambiguous 5SS and 3SS header patterns more specific and concrete.

## 1. Introduction

**Figure 1:**
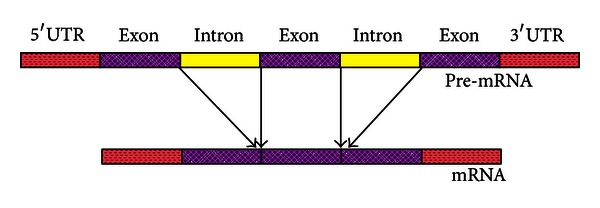
An illustration of pre-mRNA to mature mRNA.

Pre-mRNA splicing was a critical event in gene-expression pathways and mainly involved in intron removing [1]. Introns were noncoding segments in gene sequences conjoined with the protein-coding exons at splicing sites (see Figure 1). Identifying introns was the foundations for predicting the gene's structures and functions; therefore, predicting introns effectively and precisely would provide great helps in uncovering the secrets of genes [2]. Intron splicing accomplished by the spliceosome is closely related with four *cis-acting* elements, that is, the 5′ splicing sites (5SS), the 3′ splicing sites (3SS), the poly-pyrimidine tract (PPT), and the branch point (BP) [3]. Intron identification and qualification heavily depend on the four splicing signals, and, consequently, intronic sequence patterns are crucial in intron-related researches, especially in predicting the 5SS and 3SS.

Some efforts have been devoted to specifying sequence features of introns, and conceptual information such as bimodal GC% distribution [4], statistical features [5], and motifs [6] were found, but these patterns lacked concrete and specific descriptions, thereby making them hard to be used as basis of computational predictions and analyses. One more thing should be noticed is that the above-discovered patterns were all *statistically* significant only, and prejudging weights without testing the effectiveness might take a lot of risks in biased decisions. If going one step further to make the patterns *biologically* significant, it would be very inspiring.

The essentials comprising patterns were seriously explored and termed computational concerns. Three computational concerns were firstly identified as expressions, locations, and ranges. Expressions are the representations of patterns such as consensus, locations are start positions in sequences, and ranges are their possible lengths. Furthermore, for discovering biologically meaningful patterns, the *weight* concern was proposed for specifying the biological significance.

**Figure 2:**
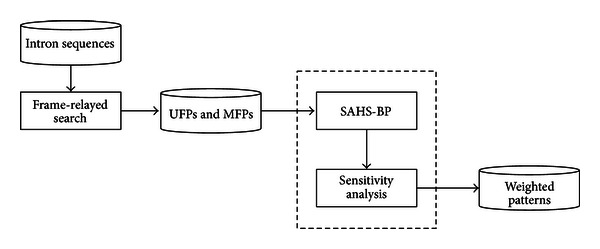
Procedure for discovering weighted patterns.

In this paper, patterns with four concerns were termed the weighted patterns. A postjudged weights discovering the methodology using hybrid self-adaptive harmony search (SAHS) and back-propagation (BP) algorithms were devised and implemented to fulfill the idea of weighted patterns. The entire processes of discovering weighted patterns were fulfilled through a frame-relayed search method [7] together with a hybrid SAHS-BP and sensitivity analysis as depicted in Figure 2.

## 2. SAHS-BP and Sensitive Analysis

**Figure 3:**
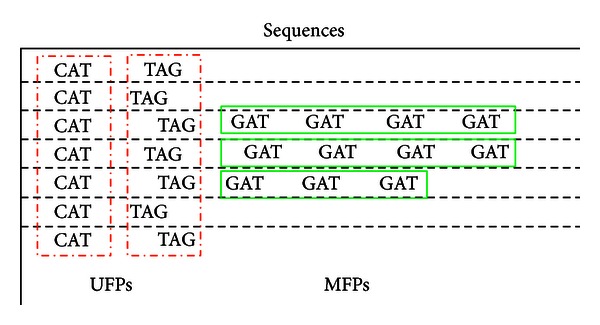
Tandem repeats of condons from the UFPs and MFPs.

In [8], Liou and Huang divided the intronic sequence features (ISF) into two categories: the uniframe pattern (UFP) and the multiframe pattern (MFP), where UFPs are the intraframe patterns and MFPs are the interframe patterns. Based on their frequencies and distributions, the significant UFPs focus on vertical distributions of tandem repeats, and the significant MFPs focus on horizontal ones, as shown in Figure 3. For detailed discussions on intronic sequence features and frame-relayed search method, see [7, 8].

After obtaining the patterns by frame-relayed search method [7], their relative importance could be derived from a new hybrid SAHS-BP mining system. The basic idea is to extract the instinct relationships between the input attributes and the output responses from the trained network by means of a postsensitivity analysis. Subsequently, the relative importance of input attributes could be determined according to these relationships. Thus, the quality of the relative importance is highly dependent on the network.

### 2.1. Hybrid SAHS-BP

**Figure 4:**
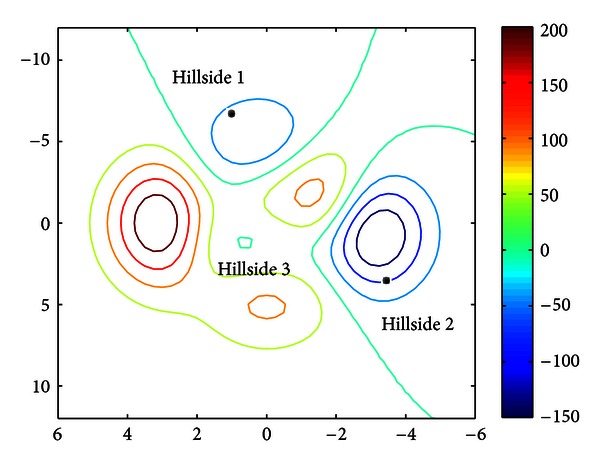
Optimization surface.

Artificial neural networks (ANN) are robust and general methods for function approximation, prediction, and classification tasks. The superior performance and generalization capabilities of ANN have attracted much attention in the past thirty years. Back-propagation (BP) algorithm [9] (i.e., the most famous learning algorithm of MLP) has been successfully applied in many practical problems. However, the random initialization mechanism of ANN might cause the optimum search process (the learning problem can be though as search through hypotheses for the one best fit the training instances [10]) to fail and return an unsatisfied solution, since the back-propagation is a local search learning algorithm [11]. For example, once the random initialization of the synaptic weights led to the search process start from hillside 1 as shown in Figure 4, BP algorithm would update the synaptic weights and go along the gradient direction. Consequently, it seems hopeless to reach a better solution near the global optimum in valley 2. Therefore, lots of trials and errors were the general guideline in most practical usage.

On the other hand, a new metaheuristic optimization algorithm-harmony search (HS) with continuous design variables was proposed recently [12]. This algorithm is conceptualized using the musical improvisation process of searching for a perfect state of harmony. Harmony search exhibits a nice global search property and seldom falls into a trap. Moreover, the HS has been successfully applied to several real-world optimization problems [13]. A recently developed variant of HS, called the self-adaptive harmony search (SAHS) [14], used the consciousness (i.e., harmony memory) to automatically adjust its parameter values. The self-adaptive mechanism not only alleviates difficulties of parameter setting but also enhances precision of solutions.

According to these observations, we are motivated to combine the advantages of SAHS and BP together and complement their own weaknesses. SAHS is used as an initializer of the neural network, that is, the generator of initial synaptic weights of BP. In other words, the lowest valley in Figure 4 is first found by SAHS; then a gradient descent-based ANN would go down carefully to obtain a precise solution. Finally, a sensitivity analysis was conducted on the well-trained network to estimate the relative importance of input attributes.

### 2.2. Sensitivity Analysis

Sensitivity analysis is a common technique to realize the relationships between input variables and output variables. It could be used to check the quality of a hypothesis model as well. The basic idea behind sensitivity analysis is to slightly alter the input variables, and then the corresponding responses with respect to the original ones would reveal the significance of the variables. Therefore, the most important part of sensitivity analysis is to determine the adequate measurements as disturbance of input variables. Although applying sensitivity analysis to neural networks had been studied in some works [15, 16], their purposes were usually identifying important factors only, while we go one step further, in this work, not only significant input attributes will be recognized but also the relative important of them will be estimated. We proposed a new measurement, disturbance, for the relative sensitivity.

**Definition 1**

The elements of disturbance instances used in the sensitivity analysis are defined as follows:

(1) xm={(1⊗d)×xm,if  m=j,xm,otherwise, ∀xm∈xj↑i,

where *x*_*j*↑_^*i*^ is the *i*th instance in the training set, with the *j*th attribute *increased* according to the disturbance ratio *d*; that is, the symbol ⊗ denotes a plus sign. In other words, except the *j*th attribute, all other attributes of the *i*th instance are fixed. Similarly, *x*_*j*↓_^*i*^ is with the *j*th attribute *decreased*; that is, the symbol ⊗ denotes a minus sign.

**Definition 2**

The relative sensitivity of *j*th attribute is defined as follows:

(2) rsj=(∑i=1p(|net(xj↑i)−net(xji)|+|net(xj↓i)−net(xji)|))×(min⁡j⁡{∑i=1p(|net(xj↑i)−net(xji)|      +|net(xj↓i)−net(xji)|)})−1,

where function net is the trained network, and the relative sensitivity is normalized by the minimal sensitivity attribute among all attributes.

## 3. Experiments

### 3.1. Data Sets

Since the lengths of introns are varied violently, for determining an adequate sequence length for pattern discovery, a pilot study on sequence compositions of introns is performed (data not shown here). As a result, we found that introns are very different from random sequences around 97 bps in the flanking regions of 5SS and 3SS. Therefore, we defined position 97 as the start position of the last frame, and then the final sequence length in the data sets would be 101 bps. For the completeness of analysis, all introns in human chromosome 1 (NCBI human genome build 36.2) were extracted, and the final data set comprised 22,448 sequences.

### 3.2. Weighted UFPs and MFPs

The weighted UFPs and MFPs discovered by the proposed SAHS-BP mining system and sensitivity analysis are listed in Tables 1 and 2, respectively. To verify the effectiveness of these weighted codons for qualifying human introns, a two-layer classifier was constructed to test the significance of these weights.

### 3.3. Two-Layered Classifier

In order to reveal the strength of discovered weighted patterns, a simple two-layered lazy classifier was constructed. The well-known nearest neighbor classifier was adopted as the based classifier due to its simplicity and efficiency. In contrast to an eager classifier, the lazy nearest neighbor classifier only memorizes the entire training instances in the training phase and then classifies the testing instances based on the class labels of their neighbors in the testing phase. In other words, the basic idea behind the nearest neighbor classifier is well explained by the famous idiom “Birds of a feather flock together.”

The Euclidean distance is the original proximity measure between a test instance and a training instance used in the nearest neighbor classifier. A weighted Euclidean distance could be extended as *d*(*x*, *x*′) = *sqrt*(∑_*i*=1_^*n*^ *wi*(*xi* − *x*_*i*_′)^2^), where *n* is the number of dimensions and *w*_*i*_, *x*_*i*_, and *x*_*i*_′ are the *i*th attribute of weight vector *w*, training instance *x*, and test instance *x*′, respectively.

**Figure 5:**
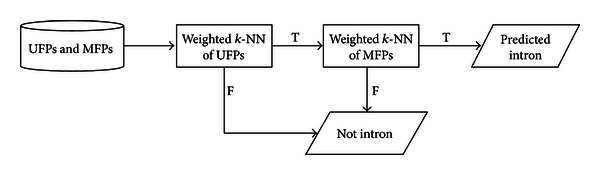
Two-layered nearest neighbor classifier.

The experiment was carried out with the 10-fold cross-validation for each specific *k* (i.e., the *k* closest neighbor). First, the whole sequence was randomly divided into 10 divisions with the equal size. The class in each division was represented in nearly the same proportion as that in the whole data set. Then, each division was held out in turn and the remaining nine-tenths were directly fed into the two-layered nearest neighbor classifier as the training instances. Since every sequence could be expressed as two parts (i.e., uniframe patterns and multiframe patterns), the first layered nearest neighbor classifier filtered out those non-intron candidates based on the weighted uniframe patterns. Finally, the prediction was made by the second layered nearest neighbor based on the weighted multiframe patterns. The flowchart of two-layered nearest neighbor classifier is shown in Figure 5.

### 3.4. Numerical Results

In this subsection, the performance comparisons between the weighted *k*-NN classifier and the conventional one are presented. Although no explicit weight vectors were used in the conventional *k*-NN classifier, the Euclidean distance indirectly implied the same importance of all input attributes. Here, we used identity vectors (i.e., all elements in the vector are one) as its weight vectors and conducted the experiment in the same process as shown in Figure 5 for the performance comparisons. The reported values of performance evaluation measures here are the averages from the 10-fold cross-validation.

**Figure 6:**
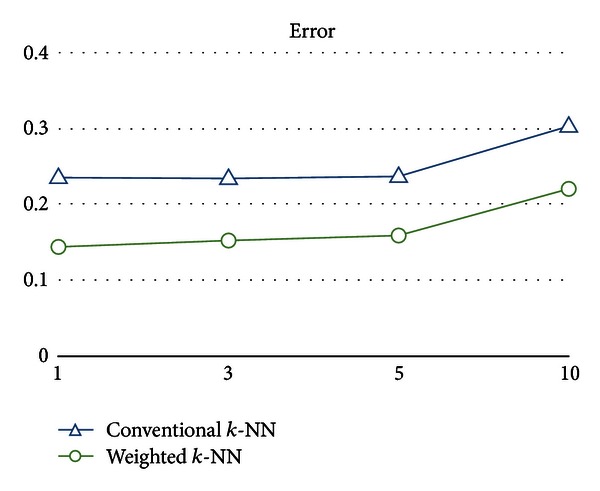
Error of the conventional *k*-NN classifier and the weighted *k*-NN classifier on different *k*.

**Figure 7:**
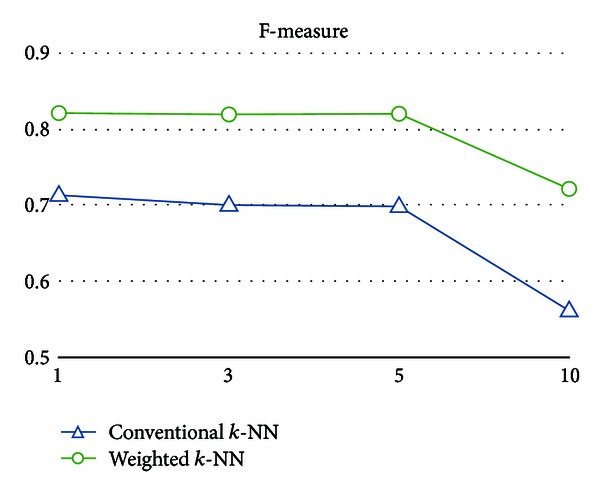
F-measure of the conventional *k*-NN classifier and the weighted *k*-NN classifier on different *k*.

**Figure 8:**
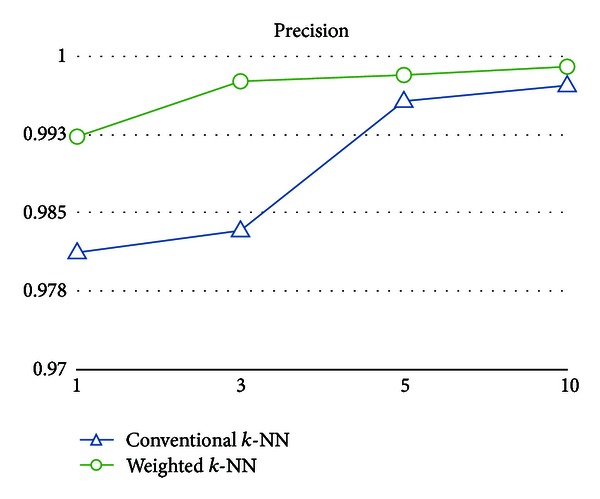
Precision of the conventional *k*-NN classifier and the weighted *k*-NN classifier on different *k*.

**Figure 9:**
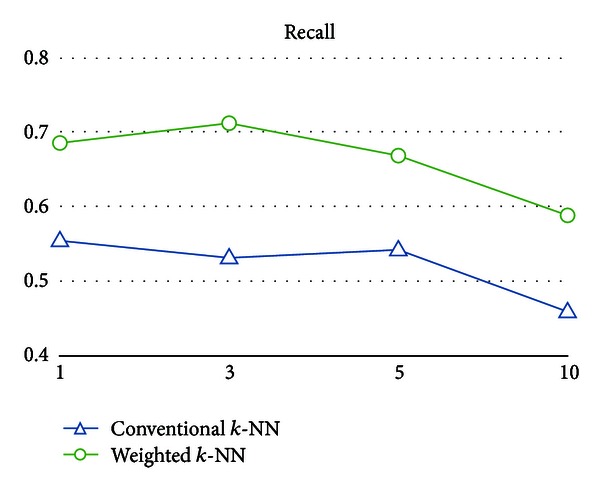
Recall of the conventional *k*-NN classifier and the weighted *k*-NN classifier on different *k*.

As shown in Figures 6, 7, 8, and 9, the numerical results clearly indicate that the weighted *k*-NN classifier performs much better than the conventional one in terms of error, F-measure, and the recall on different *k*, except precision. In addition to error decreased from 25.21% to 16.88% on average, F-measure (or recall) is also increased 12.73% (or 14.21%), respectively. From the perspective of *k* value used in *k*-NN, slightly better numeric results could be obtained from both weighted and conventional nearest neighbor classifiers for *k* = 3. Furthermore, one might argue that both weighted and conventional *k*-NN achieve such high scores in precision and relatively low scores in recall; that is, there are few predicted false positives and lots of predicted false negatives in both models. However, we believe that the reason for this circumstance is due to the inherent model bias and lazy characteristics of the nearest neighbor method. It lacks the ability to well describe the learning concept because the basic idea is merely distance comparisons. Nevertheless, such a simple weak classifier is appropriate to demonstrate the effectiveness of the weighted patterns.

Besides, since a limited number of samples were used to compare the performances of two models, we want to know whether the better performance of the weighted *k*-NN classifier is just as a result of the chance effects in the estimation process (i.e., the 10-fold cross-validation). More precisely, we should determine whether the observed difference of performance measures between two classifiers is really statistically significant (i.e., significantly better). Therefore, we used a paired *t*-test [17] on the weighted *k*-NN classifier and the conventional one with a 95% confidence coefficient.  Table 3 reveals that the weight vectors not only significantly reduce the classification error of simple nearest neighbor classifiers but also significantly improve recall and F-measure. In other words, the predicted true positives are enhanced, and the false negatives are reduced as well. Thus, we could claim that some meaningful characteristics for intron identification are really enclosed in the weighted patterns.

## 4. Discussions

Intron identification played a key role in gene-expression researches, and pattern recognition was the basis for computationally predicting exon-intron junction sites. For discovering biologically meaningful patterns in introns, three computational concerns (pattern representation, position in sequences, and the spread range of patterns) were firstly identified by frame-relayed search method [7]. After that, a hybrid self-adaptive harmony search (SAHS) and back-propagation (BP) mining system was devised and implemented to fulfill the idea of mining weighted patterns. The weighted patterns clearly provide more specific and concrete information about introns. Thus, they should be of potentials in promoting the progress of gene analyses, providing great helps in discriminating authentic splicing sites from fictitious ones and revealing the visions of *in silico* validation of intron candidates.

## Figures and Tables

**Table 1:** UFPs of 5SS and 3SS.

ID	Weight	Expression	Location	Range
5SS				

1	5.30	AAG	1	5
2	2.01	GAG	1	5
3	3.98	GTA	1	5
4	2.02	TAA	1	5
5	2.32	TGA	1	5
6	4.54	AGT	4	5

3SS				

7	3.12	ACA	1	5
8	6.26	CAG	1	5
9	1.00	CCA	1	5
10	1.09	GCA	1	5
11	2.69	TAG	1	5
12	2.33	TCA	1	5

**Table 2:** MFPs of 5SS and 3SS.

ID	Weight	Expression	Location	Range
5SS				

1	2.15	TTT	7	5
2	1.23	TGG	7	5
3	3.13	GGG	7	5
4	1.00	CTG	7	5
5	6.49	TTT	10	17
6	2.25	TGG	10	17
7	3.42	TCT	10	17
8	8.45	GGG	10	17
9	4.52	CTG	10	17
10	23.76	TTT	25	68
11	5.90	TGG	25	68
12	9.66	TCT	25	68
13	34.39	GGG	25	68
14	11.63	CTG	25	68
15	17.72	AAA	25	68
16	7.11	TTT	91	11
17	2.02	TCT	91	11
18	4.18	GGG	91	11
19	3.23	CTG	91	11
20	3.65	AAA	91	11

3SS				

21	7.63	TTT	4	5
22	5.08	TCT	5	5
23	33.13	TTT	7	17
24	11.63	TGT	7	17
25	25.46	TCT	7	17
25	3.80	CTG	7	17
27	3.20	ATT	7	17
28	20.08	TTT	22	47
29	7.39	TGT	22	47
30	12.52	TCT	22	47
31	9.78	CTG	22	47
32	7.07	ATT	22	47
33	17.48	AAA	22	47
34	2.11	TTT	67	8
35	1.76	TCT	67	8
36	2.28	CTG	67	8
37	1.89	ATT	67	8
38	2.25	AAA	67	8
39	1.51	TTT	75	5
40	1.27	CTG	75	5
41	1.02	ATT	75	5
42	1.23	AAA	75	5
43	8.31	TTT	76	26
44	3.41	ATT	76	26
45	9.53	AAA	76	26

**Table 3:** The *P* values of the *t*-test on the weighted and conventional *k*-NN classifiers.

#*k*	Measure			
	Error	F-measure	Precision	Recall
1	2.35*E* − 09	4.58*E* − 09	1.23*E* − 05	2.13*E* − 09
3	7.64*E* − 08	5.67*E* − 09	1.89*E* − 05	4.23*E* − 07
5	1.87*E* − 09	3.56*E* − 08	0.09	3.87*E* − 08
10	6.43*E* − 08	2.33*E* − 10	0.87	7.56*E* − 09
